# Growing Potatoes by Galvanism

**Published:** 1845-06

**Authors:** 


*Growing Potatoes by Galvanism.*—On the 2d July, Mr. Wm. Ross presented to the New York Farmers' Club, some potatoes measuring seven inches in circumference. He planted the seed potatoes in drills on the 6th May last, using only leaves for manure. Across three rows at one end he buried a sheet of copper 5 feet long and 14 inches wide, and at the other end, 200 feet distant, a sheet of zinc of like dimensions. The sheets were placed in an upright position, and were connected by a copper wire, thus making a galvanic battery, the moisture of the earth completing the circuit. On the 15th, some potatoes were taken from these rows, varying from one inch to one inch and a quarter in diameter. On the 2nd July, others were dug that measured 2½ inches in diameter. Some of the adjoining rows beyond the battery, were tried, but few of them had potatoes larger than marrow-fat pease—certainly none larger than a boy's marble. Mr. Ross had made other experiments with electricity from a common Leyden jar, and by applying the charges three times, he succeeded in producing cucumbers in the open ground, which measured five inches in length in thirty-seven days from the time of planting the seed.—*Mech. Mag.*

